# Screening and characterization of a denitrifying *Chelatococcus* with strong desulfurization capacity

**DOI:** 10.1016/j.heliyon.2024.e25135

**Published:** 2024-01-26

**Authors:** Chunan Du, Xiaolong Zhao

**Affiliations:** a College of Chemical Engineering, Shandong Institute of Petroleumand Chemical Technology, Dongying, 257000, China; b China University of Petroleum–Beijing at Karamay, Karamay, 834000, China

**Keywords:** Separation and identification, Denitrifying bacteria, Bioinhibition, Desulfurization

## Abstract

A denitrifying bacteria, which we named *Chelatococcus* DNB-15, was screened and isolated from the Shengli Oilfield polymer-containing wastewater. The strain was characterized by 16S rDNA analysis and the effects of nutrient substrate type, pH, mineralization and temperature on the activity of the strain were also investigated. The strain was identified as *Chelatococcus*, of which the most efficient carbon source is sodium citrate, the most suitable nitrogen source is potassium nitrate, the suitable temperature for growth is 35～45 °C, the suitable pH range for growth is 7.0～9.0, and the maximal tolerable mineralization is 4 × 10^4^ mg/L. The desulfurization experiment showed that *Chelatococcus* DNB-15 has desulfurization ability to some extent. When the initial sulfide concentration is lower than 50 mg/L, *Chelatococcus* DNB-15 grows rapidly, and the sulfides are removed quickly within 24 h, displaying a sulfur removal rate higher than 99 %; When the initial sulfide concentration is higher than 150 mg/L, the growth of *Chelatococcus* DNB-15 is severely restricted, the desulfurization effect is not obvious, and the desulfurization rate is almost stagnant.

## 1 Introduction

Sulfate-reducing bacteria (SRB) is a type of common harmful bacteria in oilfield production. They can use oil, natural gas and other organic substances as carbon sources to reduce oxidized sulfur such as SO_4_^2−^ and SO_3_^2−^ into sulfide ions [[1], [2], [3]]. The growth and reproduction of SRB and the sulfides produced by their metabolism bring many damages to the oilfield production system, including mainly the acidification of the oil reservoir, the corrosion of equipment and pipelines [[4], [5], [6]], the blockage of sulfide precipitation which affects the oil recovery ratio, and the viscosity loss of polymer solution that affects the effect of polymer flooding [[7], [8], [9]], and generation of hydrogen sulfides that are poisonous to construction workers.

At present, physical and chemical methods are mainly used to inhibit SRB in oilfields. The physical methods have a good effect on the SRB that are in free state, but are limited in the action distance, and thus these methods are incapable of killing the long-distance or attached SRB [[10], [11], [12]]. The chemical methods use common agents such as aldehydes, quaternary ammonium salts, heterocycles and their complexes [[13], [14], [15]]. The disadvantages of these chemical methods are that SRBs develop some resistance when these chemicals are used over a long period of time and that SRBs are able to produce a protective biofilm on the surface that makes it difficult for the chemical agent to penetrate. Moreover, the toxicity of the chemical agents would bring about potential environmental pollution [16,17]. The use of biological methods to inhibit SRB is a new technology developed in recent years. This new technology does not follow the traditional thinking mode of completely killing SRB. Instead, by injecting functional activators (nitrate, nitrite, etc.) into the system to activate the endogenous nitrate-reducing bacteria (i.e., denitrifying bacteria (DNB)) or injecting exogenous DNB, these DNB or endogenous DNB can compete with SRB for living space and nutrient substrates. The preferential utilization of nutrient substrates by DNB results in insufficient nutrient substrates required by SRB, which limits the activity or survival of SRB and inhibits sulfide production [[18], [19], [20]]. Inhibition of SRB and prevention of acidification of oil reservoir using the biological method, which is a green technology, can suppress SRB for longer terms and eliminate hazards caused by sulfides by changing the ecosystem of the reservoir environment. A large number of laboratory studies and experiments have been carried out at home and abroad to test this biological method to explore how it could be used to control the acidification of oil reservoirs. However, due to due to the associated problems of specific microbial activity/properties and physicochemical conditions of the treatment system, such as the activatability of native DNB, the ability of SRB and NRB in utilizing hydrocarbons or volatile fatty acids contained in crude oil, the injection dose and timing of activators, there are little reported examples of successful applications [[21], [22], [23]].

In this study, we screened and isolated a denitrifying bacterium from polymer-containing wastewater of Shengli oil field. Its morphological, physiological, and biochemical characteristics were investigated. 16S rDNA analysis was performed, and the desulfurization ability of the strain was evaluated, which provided a theoretical basis for future field applications.

## 2 Materials and methods

### 2.1 Culture medium

For denitrifying bacteria enrichment and isolation medium, 2 g of KNO_3_, 1 g of K_2_HPO_4_, 1 g of KH_2_PO_4_, 0.2 g of MgSO_4_, 5 g of NaCl, 5 g of sodium citrate, 1000 mL of distilled water were mixed and pH was adjusted to 7.2. The medium was sterilized at 121 °C for 20 min.

For solid medium, agar was added to the above medium at a weight/volume ratio of 1.5 %∼2 %. Agar was dissolved by heating and the mixture was then sterilized at 121 °C for 20 min.

All reagents were analytical grade and used as received without further purification, purchased from Macklin, China.

### 2.2 Enrichment and isolation of denitrifying bacteria

A total of 10 mL of wastewater was taken and injected into a 100 mL serum bottle that had been sterilized by high-pressure steam and contained 90 mL of denitrifying bacteria enrichment medium using a sterilized syringe. The mixture was cultivated at a constant temperature of 40 °C. When bubbles were observed, or pink color was observed when the medium was checked with Griess reagent every 12 h, it indicated a positive reaction of nitrite generation was going on. At the same time, if reduced NO_3_^−^ and increased NO_2_^−^ levels were detected using the diphenylamine reagent, it indicated denitrifying bacteria were growing well. The culture medium containing the bacteria was taken and the improved double-layered dish method was used for isolation of the denitrifying bacteria.

### 2.3 Identification of the strain

The morphology and internal structures of the bacteria were investigated. Briefly, a single colony was cultured in a single plate and the morphology of the bacteria was observed under the light microscope; the bacteria from the single colony were also observed under the scanning electron microscope (SEM,TESCAN MIRA LMS, Czech).

Molecular biological identification was carried out. Briefly, DNA was extracted from the isolated bacteria and the 16SrDNA gene was amplified using the following primers:

Forward primer 27f: 5′-AGAGTTTGATCMTGGCTCAG-3′,

Reverse primer 1492r: 5′-GGCTACCTTGTTACGACTT-3’.

The PCR amplification reaction volume was 25 μL, containing 1 μL of template DNA, 0.5 μL of each primer (27f and 1492r), 0.2 μL of Taq polymerase, 2.5 μL of 10 × PCR buffer (with MgCl_2_), 2 μL of dNTPs, and 20.3 μL of ddH_2_O. The reaction conditions were as follows: pre-denaturation at 94 °C for 5 min; denaturation at 94 °C for 1 min, annealing at 55 °C for 1 min, extension at 72 °C for 1 min, for 30 cycles; extension at 72 °C for 10 min. The PCR products were detected by agarose gel electrophoresis. The target fragments were recovered, purified, and sent to Shanghai Bioengineering Company for Sanger sequencing. The 16S rDNA sequencing results were compared with known sequences in the GenBank database. The MEGA software was used for sequence alignment and a phylogenetic tree was constructed using the Neighbor-Joining method.

### 2.4 Experiment for growth characteristics of the strain

Different environmental factors, such as pH (5, 6, 7, 8, 9, 10), culture temperature (30, 35, 40, 45, 50, 55 °C), salinity (5000, 10000, 20000, 40000, 50000, 60000, 80000 mg/L), oxygen content (based on the volume of culture medium in the culture flask, for 300 mL culture flasks, 50, 100, 200, 250 and 300 mL of cultured medium was added, respectively), carbon sources (sodium citrate, sodium acetate, glucose, etc.) and nitrogen sources (peptone, potassium nitrate, urea, ammonium sulfate, etc.), were set for stationary culturing of the bacteria. UV spectrophotometry was used to measure the changes of OD_600_ under different conditions at different times to characterize the growth characteristics of the denitrifying bacteria. Sulphide determination was carried out according to (GB/T 16489 -1996). Sulphide was detected after centrifugation to remove the bacteria.

### 2.5 Evaluation of the desulfurization ability of the strain

The denitrifying bacteria culture medium was loaded into the anaerobic bottle and sterilized at 121 °C for 20 min. After cooling down, 30, 50, 70, 100, and 150 mg/L of S^2−^ were added into different bottles, respectively. 10 % of the denitrifying bacteria cultured to logarithmic growth phase were added to each bottle for stationary culturing. The culturing medium was sampled at fixed time points to detect changes in S^2−^ concentration.

## 3 Results and discussion

### 3.1 Morphologic, physiological and biochemical characteristics of *Chelatococcus* DNB-15

**Fig. 1:**
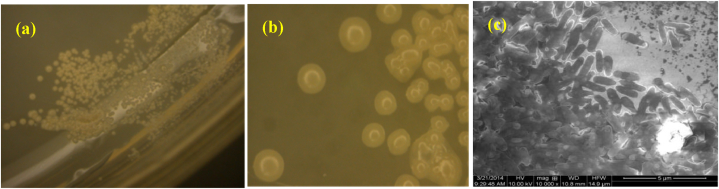
The morphological features of (a)*Chelatococcus* DNB-15 and (b) at × 6 magnification, (c) SEM of the *Chelatococcus* DNB-15.

As shown in Fig. 1a and b, the morphological features of the *Chelatococcus* DNB-15 colony were uniform, round, smooth and translucent, light yellow, and about 1–2 mm in diameter. The morphology of the *Chelatococcus* DNB-15 under the SEM is shown in Fig. 1c. Under the SEM, the *Chelatococcus* DNB-15 was short rod-shaped and about 1–2 μm long. The physiological and biochemical characteristics were shown in Table 1.

**Table 1:** The physiological and biochemical characteristics of the *Chelatococcus* DNB-15.

Identification Index	Result	Identification Index	Result
Anaerobic Growth Experiment	Growable	Methyl Red Reaction	+
Starch Hydrolase Activity	–	Mhenylalanine Deaminase Acitivity	–
Gelatin Liquefaction	–	Indole Reaction	–
Catalase Activity	+	Acetyl-Methyl-Carbinol Reaction	–
Cytochrome Oxidase Activity	+	H_2_S Production	–
Utilization of Citric Acid	+	Denitrification Reaction	+
V–P assay	–	Glucose Oxidation	Oxidized

### 3.2 Molecular biological identification of *Chelatococcus* DNB-15

**Fig. 2:**
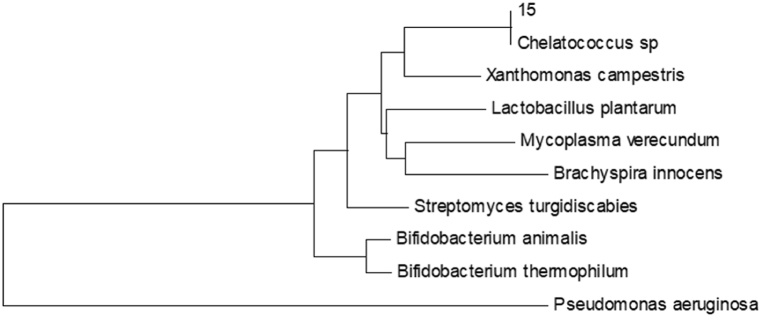
The 16S rDNA phylogenetic tree of the *Chelatococcus* DNB-15.

Similarity alignment using the GenBank database showed that the sequence of *Chelatococcus* DNB-15 was 99 % homologous to that of *Chelatococcus*. The 16S rDNA phylogenetic tree was constructed by the Neighbor-Joining method using the MEGA software, as shown in Fig. 2. Combining the strain's morphological analysis and physiological and biochemical characteristics, we determined that the strain most likely belonged to the genus *Chelatococcus*.

### 3.3 Growth characteristics of *Chelatococcus* DNB-15

**Fig. 3:**
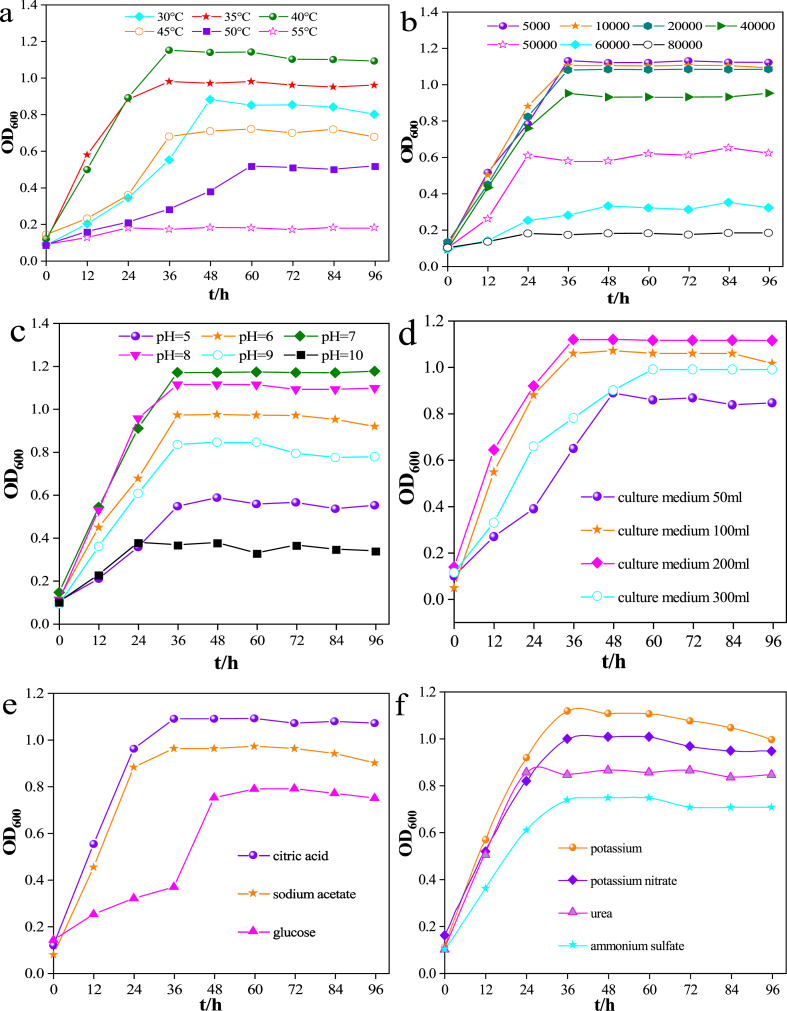
The growth status of *Chelatococcus* DNB-15 at different temperatures(a), salinity concentration(b), pH(c), oxygen concentration(d), carbon source(e) and nitrogen source(f).

From the growth status of *Chelatococcus* DNB-15 at different temperatures (Fig. 3a), we can see that DNB-51 has strong adaptability to temperature. Within 30–40 °C, as the temperature increased, the concentration of the bacteria also increased. When the temperature rises consistently to 50 °C, the growth rate of bacteria decreases significantly. When the temperature was further increased to 55 °C, the bacteria did not increase through time. This represents the inability of Chelatococcus DNB-15 to survive at temperatures above 55 °C. It might be caused by the gradual inactivation of nucleic acids, proteins and other components in the bacteria when the temperature was higher than a certain range, the enzymatic reaction could not be carried out, and the metabolic activity of the strain was reduced, which showed the inhibition of the growth of the strain.

*Chelatococcus* DNB-15 was cultured in medium containing different NaCl concentrations (0.5 %, 1 %, 2 %, 4 %, 5 %, 6 %, 8 %). The growth curves are shown in Fig. 3b. When the salinity concentration was lower than 2 %, the growth of *Chelatococcus* DNB-15 was not affected at all; When the salinity concentration was between 2 % and 4 %, the growth of *Chelatococcus* DNB-15 was not significantly affected; When the salinity concentration was between 5 % and 6 %, the growth of *Chelatococcus* DNB-15 was significantly affected; When the salinity concentration was higher than 8 %, the *Chelatococcus* DNB-15 bacteria were almost not growing. Thus, the maximal tolerable salinity of *Chelatococcus* DNB-15 was 4 %.

As shown in Fig. 3c, *Chelatococcus* DNB-15 could grow well in the pH range of 7.0–9.0. When the pH was lower than 6.0 or higher than 10.0, the growth of *Chelatococcus* DNB-15 was significantly affected. When the pH is too high or too low, macromolecules such as cellular proteins and nucleic acids will be denatured, and thus the biological activity of the bacteria will be affected. In addition, the charged properties and stability of substances inside the cell membrane will be affected, and the ability of bacteria to absorb nutrients could be affected. Moreover, pH can also affect the activity of microbial enzymes, in which case bacteria can grow but at a slow rate.

As shown in Fig. 3d, when the oxygen concentration was low, the smaller the amount of culture medium, the greater the amount of oxygen in the upper space of the culture flask, the higher the mass transfer rate, and the faster the bacterial growth. Compared with strictly anaerobic conditions, *Chelatococcus* DNB-15 is a facultative anaerobic bacteria that can obtain energy by different oxidative methods in the presence of trace amounts of oxygen, and has both aerobic respiration and anaerobic fermentation. Therefore, the presence of small amounts of oxygen can promote its reproduction.

However, when cultured in a shaking incubator for 7 days, the bacteria did not grow, and after static culture, the bacteria still did not grow, indicating that high concentrations of oxygen can severely inhibit *Chelatococcus* DNB-15 growth.

#### 3.3.1 The effect of different carbon sources on the growth of *Chelatococcus* DNB-15

As shown by the curves of DNB- 15 growth by different carbon sources (Fig. 3e), citric acid has the best effect on strain growth, followed by sodium acetate and glucose. The reason for this difference may be due to the fact that Chelatococcus DNB-15 possesses a high activity of the enzyme specifically recognised for citric acid. And citric acid can easily enter the cell for further utilization due to its small molecule nature. Nitrogen source is also an important element for bacterial growth. The experimental results show that the utilization effect of *Chelatococcus* DNB-15 on nitrogen source is best in potassium nitrate, followed by peptone, urea and ammonium sulfate (Fig. 3f). Peptone is the most important basic component of bacterial culture, which can provide abundant nutrients for the growth of most bacteria. Potassium nitrate is an inorganic nitrogen source with a single chemical composition, which is easy to be absorbed and utilized by bacteria. These two are important for *Chelatococcus* DNB-15 culturing. When ammonium sulfate is used as the nitrogen source, the pH of the system is lowered due to the physiological acidity, which negatively affects the culture effect.

### 3.4 Desulfurization ability of *Chelatococcus* DNB-15

**Fig. 4:**
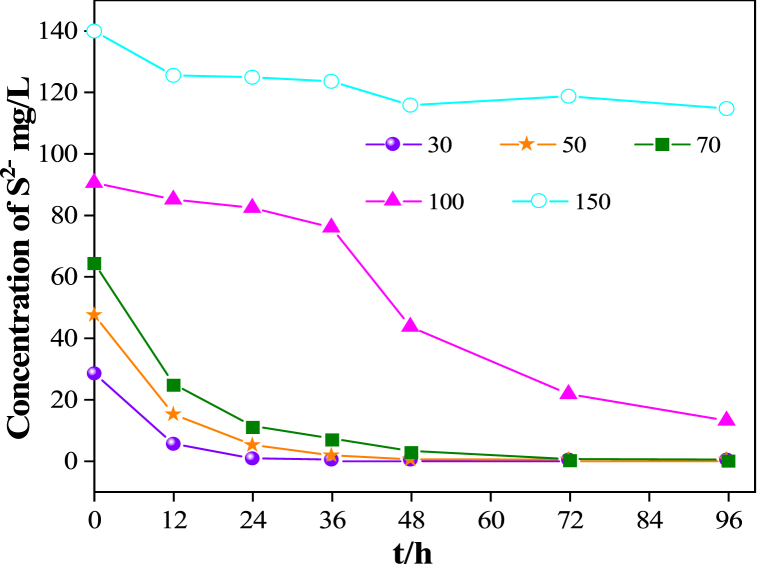
The desulfurization test of *Chelatococcus* DNB-15.

From the experimental results of the desulfurization test of *Chelatococcus* DNB-15 (Fig. 4), we can see that *Chelatococcus* DNB-15 has desulfurization ability to some extent. When the initial sulfide concentration was lower than 50 mg/L, *Chelatococcus* DNB-15 grew fast and sulfides were quickly removed within 24 h, with a sulfur removal rate of over 99 %. Moreover, sulfur precipitation could be seen at the bottom of the culture bottle, and the medium appeared milky white. With the increase of sulfide concentration, the ability of *Chelatococcus* DNB-15 in removing sodium sulfide weakened. When the initial sulfide concentration was 100 mg/L, the growth of *Chelatococcus* DNB-15 was limited, and the removal rate of sulfides in the medium slowed down. At 96 h, the removal rate of sulfides was 86.2 %. When the initial sulfide concentration reached 150 mg/L, the growth of *Chelatococcus* DNB-15 was severely restricted, the removal effect of sulfides was not obvious, and the removal rate was almost stagnant. This is because sulfides are biologically toxic and have an inhibitory effect on bacterial growth [24]. As the concentration of sulfides increases, the inhibitory effect becomes stronger.

## 4 Conclusion

A denitrifying bacterium, Chelatococcus DNB-15, was screened and isolated from polymer-containing wastewater of Shengli oilfield. This denitrifying bacterium, was able to grow and multiply rapidly at 35–45 °C and pH 7.0–9.0 when sodium citrate was used as the carbon source and potassium nitrate was used as the nitrogen source. And Chelatococcus DNB-15 has a certain degree of desulfurization ability. When the initial sulfide concentration was lower than 50 mg/L, sulfide was rapidly removed within 24 h. It provides a feasible bacterial option for biological treatment of oily wastewater for further nitrogen and sulfur removal.

## Data availability statement

The date will be made available on request.

## Additional information

No additional information is available for this paper.

## CRediT authorship contribution statement

**Chunan Du:** Writing – original draft, Software, Methodology, Investigation, Formal analysis, Data curation. **Xiaolong Zhao:** Writing – review & editing, Writing – original draft, Supervision, Resources, Methodology, Investigation, Formal analysis, Conceptualization.

## Declaration of competing interest

The authors declare that they have no known competing financial interests or personal relationships that could have appeared to influence the work reported in this paper.
